# Volume kinetic evaluation of fluid turnover after oral intake of tap water, lemonade and saline in volunteers

**DOI:** 10.1186/s13102-016-0045-x

**Published:** 2016-07-28

**Authors:** Joachim Zdolsek, Annika Metander, Robert Hahn

**Affiliations:** 1 Department of Anaesthesia and Intensive Care, Faculty of Health Sciences, Linköping University, Linköping, Sweden; 2 Department of Anaesthesiology and Intensive Care, Vrinnevi Hospital, S-603 79 Norrköping, Sweden; 3 Research Unit, Södertälje Hospital, Södertälje, Sweden

**Keywords:** Hydration, Exercise, Blood volume

## Abstract

**Background:**

Volume kinetic fluid turnover of three beverages was investigated for the purpose of estimating their rates of absorption and elimination as well as their maximum effect on the blood volume. The results were then used to simulate the effects of ingesting different combinations of these fluids.

**Method:**

Ten healthy volunteers ingested 0.5 L of tap water, lemonade (90 g/L carbohydrates) and isotonic saline (9 g/L) on different occasions. Venous blood samples for measurement of the blood haemoglobin (Hb), haematocrit and glucose concentrations were collected on 10 occasions over 2 h. A kinetic model based on haemoglobin dilution and urinary excretion was used to estimate the rate of absorption, the blood volume expansion over time, and the rate of elimination. Obtained kinetic data was used to simulate combinations of the three beverages in order to reach a predetermined goal of a 1:1 hydration of the blood volume and peripheral tissues over 6 h.

**Results:**

Tap water had the fastest absorption but primarily hydrated peripheral tissues. Maximum hydration was reached after 17 min. Lemonade effectively expanded the blood volume and was absorbed and excreted at a high rate. The maximum hydration from isotonic saline occurred 60 min after ingestion. Slow excretion could make it possible to use saline to prolong the effects of the other two beverages.

**Conclusions:**

It is possible to use the kinetic model to evaluate fluid turnover and compartmental distribution. Composition and timing of fluid intake can be calculated mathematically to meet predetermined goals of hydration and distribution.

**Trial registration:**

NCT01360333 Date of registration: 05/23/2011.

**Electronic supplementary material:**

The online version of this article (doi:10.1186/s13102-016-0045-x) contains supplementary material, which is available to authorized users.

## Background

Orally ingested fluids are absorbed and excreted differently, depending on the ingested volume, tonicity and composition of the fluid. When a certain effect is desired, the composition of the fluid has to be adjusted accordingly [1]. These effects may include treatment of dehydration, supportive treatment and hydration prior to sport activity. An important aspect of the choice of fluid is time dependency, that is, the fluid’s effect over time, which is governed by the absorption and compartmental distribution of the orally ingested fluid. Fluid kinetics of beverages has earlier been studied using single point samples, ultrasound and gastric tubes to measure gastric emptying, body weight or urine-specific gravity. However, these techniques provide only snapshots or simplified views of what happens with the fluid in the body from ingestion to excretion.

Volume kinetics is related to pharmacokinetics and has been developed to capture the effects of intravenous fluid therapy, but there is reason to believe that this methodology can be used to study oral ingestion of fluid as well. Instead of using indirect measures, such as the emptying of the ventricle, volume kinetics estimates the effects of ingested fluid on the blood volume and body fluids. This is of interest to sports medicine because the blood volume is a major determinant of cardiac output [2–8].

If the kinetics of a beverage is known, the structural parameters could possibly be used to tailor a desired effect by combining different fluids. In sports, for example such tailoring could be useful [1, 7].“Timing” of the intake is, in that case, essential. The beverage that offers optimal performance effects would contain glucose and/or salt in various combinations in order to support metabolic needs and replace fluid and salt losses by sweating. Further, the addition of sugar or salt reduces the volume of distribution of the fluid, which increases blood volume expansion.

The first aim of this study was to determine whether it is feasible to measure different effects of fluids with the volume kinetic method. The second aim was to create mathematical models that simulate what fluids and compositions are needed to achieve desired effects. The input for the analysis was serial measurements of haemodilution, which is the inverse of intravascular water volume [9, 10].

## Methods

The study was approved by the regional ethics committee in Linköping, Sweden (Dnr 2010/241-31) and registered at ClincalTrials.com with identifier NCT01360333. Data was collected during 2011. Ten healthy volunteers, seven males and three females, aged 33 ± 10 years (mean ± standard deviation [SD]; range 21–48 years) and with a body weight of 77 ± 13 kg (range 58–95 kg), were recruited for the three experiments. After being informed about the study both orally and in writing, each volunteer gave his/her consent for participation.

The participants arrived at the Department of Intensive Care at Linköping University Hospital between 7:30 and 8:30 a.m. The volunteers had fasted since midnight, but to ensure that they were euhydrated prior to the study, they were told to eat one sandwich and drink one glass (2 dL) of clear liquid shortly prior to 6:00 a.m. All participants voided prior to beginning of the study and were then randomised to ingest one of three beverages. To achieve a haemodynamic steady state, volunteers rested for 30 min before the experiments began. During this time, a cannula was placed in the cubital vein of one arm to sample blood. In every case, the first blood sample was collected more than 2 h after any previous ingestion of liquid. The volunteers rested on a bed below an OPN Thermal Ceiling radiant warmer (Aragon Medical, River Vale, NJ) placed about 1 m above them, and the heat was adjusted to achieve optimal comfort.

### Ingested fluids

Each volunteer underwent the following three experiments in random order, separated by at least seven days:

A. Tap water, 0.5 L (orally).
B. Carbohydrate-containing soft drink (90 g carbohydrates/L), 0.5 L (orally).
C. Saline, NaCl (9 g/L), 0.5 L (orally).

The tap water contained an average of 0.009 g/L of sodium, 0.002 g/L of potassium and 0.019 g/L of calcium.

### Measurements

Venous blood (1–3 mL per tube) was withdrawn from the venous cannula using a vacuum tube just before and at 5, 10, 15, 20, 30, 45, 60, 90 and 120 min after the ingestion of the beverage. The baseline sample was drawn in duplicate, and the mean was used in all calculations. Before each sampling, a small volume of blood was drawn, and the volume was replaced by 2 mL of 0.9 % saline to prevent clotting.

All blood samples were analysed in the hospital’s central laboratory. The blood haemoglobin (Hb) concentration and the haematocrit (Hct) were analysed in EDTA tubes on a Cell-Dyn Sapphire (Abbott Diagnostics, Abbott Park, IL). The coefficient of variation (CV) for these analyses, based on the duplicate baseline samples, was 0.8 %. Plasma was used for measurement of the sodium, potassium (litium-heparine plasma gel tube) and glucose concentrations (Na-fluorid/oxalat-tube) on an ADVIA^®^ 1800 Chemistry System (Siemens, Eschborn, Germany) with a CV of 1.0, 1.5 and 1.5 %, respectively. At the end of the study, all participants voided and the volume was measured.

### Calculations

The following volume kinetic calculations are derived from pharmacokinetics.

The absorption, volume of distribution and elimination of fluid were analysed by a one-volume kinetic model.

Fluid was absorbed at a rate determined by a rate constant *k*_a_ to increase the volume of central body fluid space *V* to *v*. The rate of elimination was given as the product of the dilution *V* and the clearance *CL*. The differential equation is as follows:

$$ \mathrm{d}v/dt={k}_aD{e}^{-{k}_at}{\textstyle\ \hbox{-}\ }CL\left(v{\textstyle\ \hbox{-}\ }V\right)/V $$

where *D* is the volume of ingested drink. The Hb-derived fractional plasma dilution was used to indicate the volume expansion *V* resulting from the infusion [11]:

$$ \left(v{\textstyle\ \hbox{-}\ }V\right)/V=\left(\left[\mathrm{H}\mathrm{b}/\mathrm{h}\mathrm{b}\right]\ {\textstyle \hbox{-}\ }1\right)/\left(1{\textstyle\ \hbox{-}\ }\mathrm{H}\mathrm{c}\mathrm{t}\right) $$

Symbols in capital letters denote baseline values. The minor artificial dilution due to the blood sampling was corrected mathematically [11]. Elimination of fluid by evaporation was disregarded.

The structural parameters in the model (*k*_*a*_*, V* and *CL*) were estimated using population modelling in Phoenix software for non-linear mixed effects, NLME version 1.3 (Pharsight, St Louis, MO). The possibility to stabilise the analyses by using the renal clearance *CL*_R_ as *CL* was used:

$$ CL=\frac{{\displaystyle \sum}\mathrm{urine}\ \mathrm{volume}\ }{AUC\kern0.5em for\ \left(v-V\right)/V} $$

where AUC = area under the curve. The half-life was obtained as ln 2 * *V* / *Cl*.

Simulations were performed with programs developed by us for the Matlab R2012b software (Math Works Inc., Natick, MA).

The plasma volume expansion was obtained as the product of the simulated plasma dilution, that is, ((v–V)/V) and the estimated plasma volume at baseline. The latter was obtained as (1–haematocrit) x 7 % of the body mass.

The amount of absorbed fluid that remained in the body at any time *t* was calculated as the product of the plasma dilution and *V*.

### Statistics

Data are given as the mean, or best estimate, and SD. Differences were evaluated by the Wilcoxon matched-pair test.

## Results

### Haemoglobin dilution

**Fig. 1:**
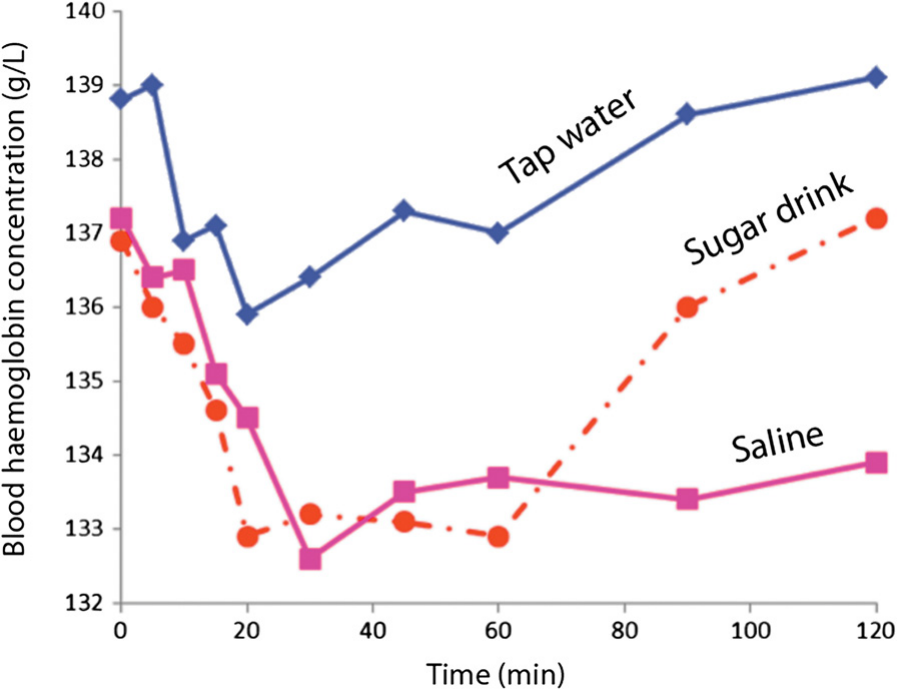
Blood haemoglobin (B-Hb) concentration over the course of the study. Crude data before recalculation to plasma dilution. Each point is the mean of 10 measurements

All 10 volunteers ingested all three fluids. Tap water caused maximal haemodilution 20 min after ingestion (*P* < 0.05). Both saline and the sugar drink were followed by more pronounced haemodilution (*P* < 0.01 at 30 min for both), and saline continued to cause haemodilution up to the study’s conclusion at 120 min (*P* < 0.01) (Table 1 and Fig. 1).

**Table 1:** Series of blood haemoglobin (B-Hb) concentrations, baseline haematocrit (Hct) and baseline concentrations of serum sodium (S-Na), plasma glucose (P-glucose) and serum potassium (S-K). All values are presented as mean ± SD

	Tap water	Sugar drink (mean ± SD)	Saline
Time series			
B-Hb (g/L), 0 min	138.8 ± 10.9	136.9 ± 14.3	137.2 ± 14.0
5 min	139.0 ± 10.2	136.0 ± 13.8	136.4 ± 14.5
10 min	136.9 ± 11.3	135.5 ± 13.9	136.5 ± 13.9
15 min	137.1 ± 10.7	134.6 ± 14.1	135.1 ± 13.3
20 min	135.9 ± 11.5	132.9 ± 13.7	134.5 ± 13.7
30 min	136.4 ± 11.3	133.2 ± 14.7	132.6 ± 12.6
45 min	137.3 ± 11.0	133.1 ± 14.8	133.5 ± 13.5
60 min	137.0 ± 12.1	132.9 ± 15.7	133.7 ± 14.2
90 min	138.6 ± 11.4	136.0 ± 14.0	133.4 ± 12.5
120 min	139.1 ± 10.9	137.2 ± 13.2	133.9 ± 12.7
Baseline			
Hct (%)	40.9 ± 3.5	40.4 ± 4.2	40.2 ± 3.5
S-Na (mmol/L)	140.5 ± 1.7	139.7 ± 2.1	140.5 ± 1.6
S-K (mmol/L)	3.75 ± 0.21	3.70 ± 0.18	3.68 ± 0.23
P-glucose (mmol/L)	5.46 ± 0.39	5.31 ± 0.47	5.41 ± 0.49

### Fluid balance

**Fig. 2:**
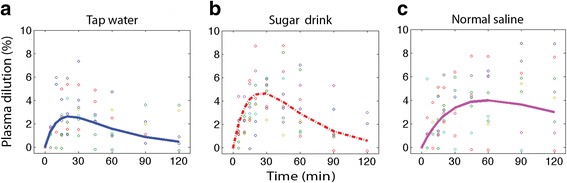
Dilution of venous plasma after ingestion of three different beverages (Tap water **(a)**, Sugar drink **(b)** and Normal saline **(c)**). Each point is one estimate of plasma dilution, and the solid line is the modelled average curve

**Fig. 3:**
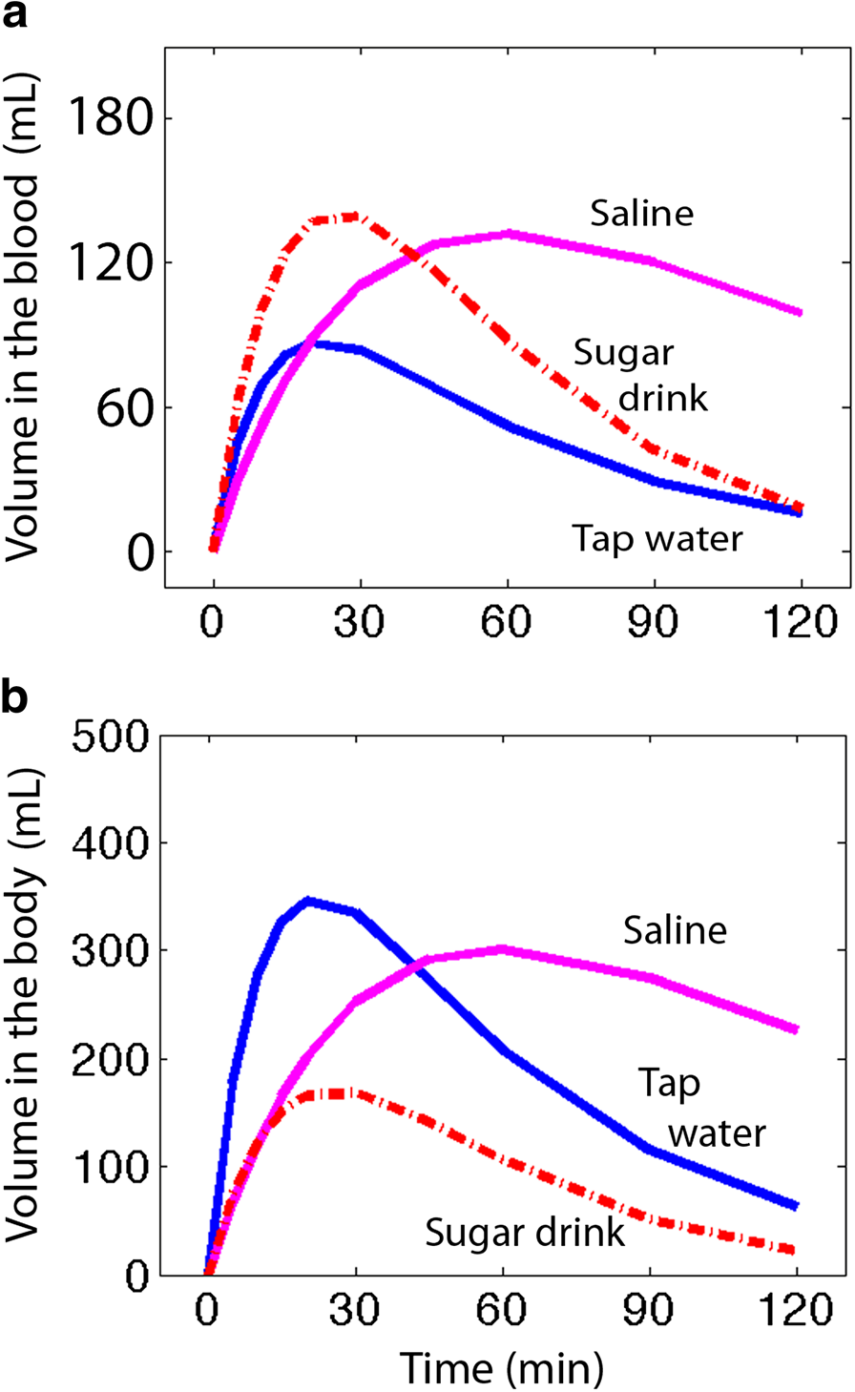
Modelled blood volume expansion (**a**) and volume of infused fluid remaining in the body (**b**) after ingestion of three different beverages

Results of the kinetic analysis are shown in Table 2. The differences between the ingested water volumes with regard to the rates of uptake and elimination are illustrated in Figs. 2 and 3. Tap water was absorbed at the highest rate but caused the least pronounced expansion of the blood volume. Most of the absorbed sugar drink volume remained in the blood, and minimal amounts were distributed to the tissues. The blood volume expansion yielded by saline was equally as pronounced as that yielded by the sugar drink, but it was more prolonged.

**Table 2:** Kinetic constants derived from kinetic analysis of the water volume content of three beverages taken by mouth on different occasions by 10 volunteers

	Tap water		Sugar drink		Saline	
	Best estimate (SD)	CV%	Best estimate (SD)	CV%	Best estimate (SD)	CV%
*k*_a_ (10^−3^ min^−1^)	51 ± 9	18	43 ± 4	10	16 ± 2	13
V (L)	5.7 ± 0.6	11	4.4 ± 0.6	14	4.2 ± 1.0	25
CL (mL min^−1^)	291 ± 58	20	190 ± 24	13	69 ± 15	21
t lag (min)	7.9 ± 2.0		7.8 ± 1.0	13	not significant	---

*k*_*a*_ absorption rate constant, *V* volume of distribution, *CL* clearance

Excretion of the ingested volume was fastest for tap water, intermediate for the sugar drink and slowest for saline. Their average half-lives were 13, 16 and 43 min, respectively.

Urinary excretion during the study period differed considerably between the three fluids, the most substantial being after ingestion of the sugar drink (567 ± 207 mL) and tap water (505 ± 194 mL). Both were significantly higher (*P* < 0.05) than excretion after the ingestion of saline, after which the urine volume amounted to only 266 ± 144 mL.

### Glucose and electrolytes

The baseline concentrations of the serum sodium and potassium and the plasma glucose are shown at the bottom of Table 1.

**Fig. 4:**
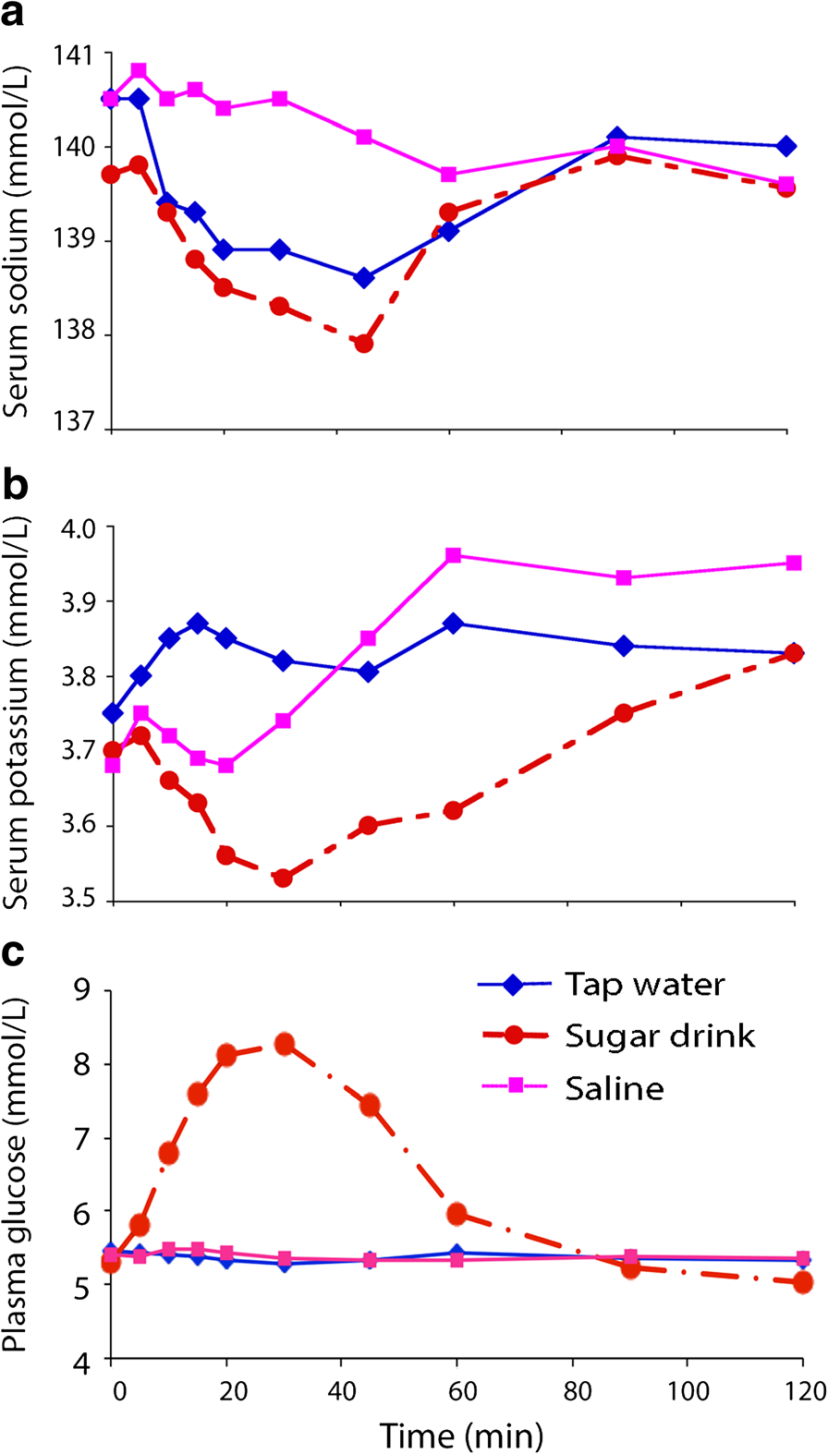
Serum sodium (**a**), serum potassium (**b**) and plasma glucose (**c**) concentrations after ingestion of three different beverages. Each point is the mean of 10 measurements

The serum sodium concentration decreased after ingestion of both tap water (0 min vs 50 min; *P* < 0.01) and the sugar drink (*P* < 0.01; Fig. 4a).

Serum potassium increased slightly after tap water ingestion, but decreased after ingestion of the sugar drink (0 min vs 30 min; *P* < 0.05). Saline ingestion increased serum potassium from 3.68 ± 0.23 at baseline to 3.96 ± 0.31 mmol/L after 1 h (*P* < 0.05; Fig. 4b).

Blood glucose rose quickly from 5.3 ± 0.5 to a maximum of 8.3 ± 1.3 mmol/L at 30 min after ingestion of lemonade (*P* < 0.01; Fig. 4c). Blood glucose did not change in response to ingestion of the other two beverages.

### Simulations

Computer simulations were performed with the aim of finding a pattern of fluid intake and a mixture of the three beverages that maintained a stable and marked blood volume expansion of up to 420 min (and also to hydrated, but not excessively, the peripheral tissues). A proportion of 1:1 between the blood and peripheral tissues was considered adequate.

**Fig. 5:**
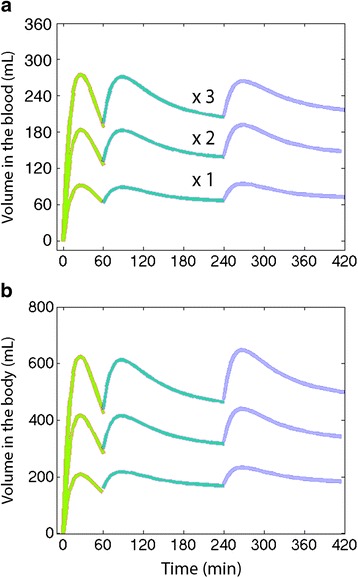
Blood volume expansion (**a**) and volume of infused fluid remaining in the body (**b**) when three beverages are combined to create a stable expansion of the blood and peripheral tissues in the approximate proportion 1:1. A low total (x1) and multiples thereof are shown. Computer simulation based on the kinetic constants from Table 2. Three intakes or doses (0, 60 and 240 min) of different fluid combinations and volumes are simulated up to 420 min. The first dose requires a different composition than the other two

To quickly hydrate both body fluid volumes, a combination of tap water and the sugar drink was found to be most effective. Later fluid intake should contain less volume but relatively more saline to prolong blood volume expansion (Fig. 5).

## Discussion

The kinetic analysis yielded the rates of uptake and elimination as well as the volume of distribution for the three liquids. The kinetic profiles of the tested fluids proved to be quite different, which is of interest for the timing and dosing of beverages [12]. Tap water was quickly absorbed but primarily hydrated the peripheral tissues, whereas the glucose drink more effectively expanded the blood volume. The downside of both of these fluids is that they were excreted quickly. Saline can be used to prolong the volume effect, as saline distributes to both the blood and to peripheral tissues, but is excreted more slowly.

Previous research contains some contradiction between the rate of gastric emptying and effect on the blood volume [13, 14]. Ingesting carbohydrate-rich fluid before a surgical procedure, in contrast to fasting, improves postoperative bowel function [15]. In the present study, the subjects were told to drink 2 dL of clear fluid and eat one sandwich 2 h prior to the beginning of the study. This may explain why the gastric emptying and uptake of carbohydrate fluid occurred quickly. Because the bowel was stimulated with fluid and food, it was better prepared to handle the carbohydrate fluid ingested during the study. Further, the subjects were at rest, thus optimising bowel function.

The computer simulation in Fig. 5 suggests how beverages may be combined to reach predefined goals. We assumed that increasing blood volume, which typically places an athlete higher on the Frank-Starling curve, would be such a goal. Pre-exercise hyperhydration [7, 16] reduces heart rate during exercise, indicating larger preload as well as stroke volume. Hydration of peripheral tissues for oxygenation and temperature regulation is also needed [7, 17, 18], but the focus would still fall primarily on expanding blood volume. In such simulations one may further consider losses of salt, water and glucose to make the situation more realistic with regard to various exercises, but such adaptations were not performed in this study.

The simulations highlight that a substantial amount of fluid must be ingested to expand the blood volume. To increase the blood volume by 400 mL within 30 min, one could ingest 900 mL of mostly lemonade and tap water with the addition of a small amount of saline, a combination that results in a glucose concentration of 4 %. The amount of further intake required to maintain a steady state is much lower. However, because our data are valid only for volunteers at rest, Fig. 5 should not be applied directly to athletes performing strenuous exercise.

The concepts used in the calculations were derived from pharmacokinetics, but their application to fluid volumes has been refined in anaesthesia research [19]. Haemodilution is calculated by a series of measurements of Hb that, together with the measured urine volume, is entered into a set of differential equations that include the unknown parameters in a kinetic model. In the present study, the three unknown parameters represented the rate of fluid uptake, the volume of distribution at baseline and the rate of elimination. The calculations of water volume distribution account only for the volume absorbed by the mechanism *k*_a_. Therefore, the fluid volume that remains in the gut at any time *t* is 500 ml minus the absorption function, which is the first part of the differential equation used to estimate the kinetic parameters. However, our calculations assumed complete absorption of the ingested water at the end of the 2-h period of the study.

The beverages caused changes in the blood concentration of some small ions. As expected, serum sodium decreased after ingestion of the two sodium-free fluids, and blood glucose increased markedly after intake of lemonade. The decrease in serum potassium following ingestion of lemonade is probably a co-phenomenon with the uptake of glucose by the body cells. Even though saline contains no potassium, it raised the serum potassium concentration. The reason for this effect is that isotonic saline contains 50 % more chloride ions than the extracellular fluid, which causes acidosis, which in turn promotes potassium translocation from the cells. The slow excretion of saline as compared to tap water and lemonade can be explained by a reduction of the glomerular filtration rate by 10 %–15 % [20] and the fact that the kidneys must concentrate the urine to excrete the sodium load [21]. The prolonged hydration capacity and content of salt makes saline a useful beverage in warm environments [1, 18].

The limitations of the present study include the low level of inflicted haemodilution, which increases the impact of sampling technique and measurement errors on the precision of the kinetic parameters. Therefore, a commonly used technique of pooling all Hb values for each fluid (the naïve approach) was used for the kinetic evaluations. The presented kinetic parameters do not capture between-subject variability but provide a representative picture of the overall effects of the beverages. To provide suggestions about the use of the three beverages during physical exercise, data should be collected in that setting.

## Conclusions

It is possible to use the volume kinetic model to evaluate the fluid turnover and compartmental distribution of oral liquids on a group basis. The composition and timing of fluid intake can be calculated mathematically to meet predetermined goals of hydration and water volume distribution. If a rapid effect of short duration is desired, water or a carbohydrate-rich beverage should be chosen. If a more long-lasting effect is desired, beverages containing sodium should be used [4, 22, 23]. Further studies will focus on optimising the beverages with respect to exercise tasks.

## Additional file

Additional file 1: Complete laboratory results. (XLS 48 kb)
